# True Strength of Ceramic Fiber Bundles: Experiments and Simulations

**DOI:** 10.3390/ma14010064

**Published:** 2020-12-25

**Authors:** Leandro Neckel, Cristian A. Faller, Matej Babič, Oscar R. K. Montedo, Carlos P. Bergmann, Rolf Janssen

**Affiliations:** 1 Programa de Pós-Graduação em Engenharia de Minas, Metalúrgica e de Materiais—PPGE3M, Universidade Federal do Rio Grande do Sul—UFRGS, Av. Osvaldo Aranha, 99. Sala 711, 90035-190 Porto Alegre, Brazil; bergmann@ufrgs.br; 2 Grupo de Pesquisa em Cerâmica Técnica—CERTEC, Universidade do Extremo Sul Catarinense—UNESC, Av. Universitária, 1105-P.O. Box 3167, 88806-000 Criciúma, Brazil; cristian.faller@hotmail.com (C.A.F.); oscar.rkm@gmail.com (O.R.K.M.); 3 Laboratory for Complex Systems and Data Sciences, Faculty of Information Studies, Ljubljanska cesta 31a, 8000 Novo Mesto, Slovenia; babicster@gmail.com; 4 Institute of Advanced Ceramics, Hamburg University of Technology—TUHH, Denickestrasse 15, D-21073 Hamburg, Germany

**Keywords:** alumina fiber, fiber bundle, load sharing, tensile behavior, simulation

## Abstract

A study on the strength of ceramic fiber bundles based on experimental and computational procedures is presented. Tests were performed on single filaments and bundles composed of two fibers with different nominal fiber counts. A method based on fiber rupture signals was developed to estimate the amount of filament rupture during the test. Through this method, the fiber bundle true strength was determined and its variation with the initial fiber count observed. By using different load-sharing models and the single filament data as input parameter, simulations were also developed to verify this behavior. Through different approaches between experiments and simulations, it was noted that the fiber bundle true strength increased with the fiber count. Moreover, a variation of the fibers’ final proportion in the bundles relative to the initial amount was verified in both approaches. Finally, discussions on the influence of different load-sharing models on the results are presented.

## 1. Introduction

Fibers are used for several purposes, including structural reinforcement, thermal insulation, and electrical insulation, among others. Polycrystalline fibers based on alumina are resistant to oxidation, but have their mechanical properties strongly affected when exposed to environments with temperatures above 1200 °C. This is due to the microstructural changes that occurred under such conditions, which also facilitate the diffusion of grain contours. Combined with a second phase, such as mullite or zirconia, α-alumina fibers have their creep resistance apparently improved. However, such fibers are sensitive to alkaline contamination, leading to the growth of alumina grains under high temperatures and a consequent decrease in their mechanical strength.

In terms of application, there is a great effort to produce more powerful, lighter, and more efficient turbines in the aerospace industry. For example, it is possible to achieve greater efficiency by increasing the working temperature. For this to be possible, ceramic matrix composites are excellent candidates to replace components that do not support such oxidizing atmospheres and with such a high service temperature. Ceramic matrix composites, in which ceramic fibers are embedded in a ceramic matrix, are designed to overcome the intrinsic fragility of monolithic ceramics, also aiming at structural applications at extremely high operating temperatures. In addition, another advantage of using such materials is the reduction of weight in components, given the low density of the ceramic material compared to the metallic one, for example. In addition, ceramic matrix composites, both matrix and the reinforcement phase consisting of ceramic oxides, are highly resistant to oxidation, due to their chemical and mineralogical nature.

Some authors have devoted efforts to determine the tensile strength of fine fiber bundles using different techniques. In particular, R’Mili et al. [1] and Calard and Lamon [2] developed techniques for determining the strength of bundles with different amounts of fibers. In their work, they sought to optimize the determination of such parameters considering side-effects of sample preparation, such as the possible variation in the initial amount of fibers in each test. Thereby, fiber breakage is of a statistical nature because of the non-uniformity of fiber strength along the fiber length and the load redistribution [3]. Bundles with more fibers tend to bear more load but present lower engineering strength when compared to single filaments [4]. Experimentally, determining the engineering strength of bundles of micrometric fibers is difficult due to the measurement of the cross-section and the actual amount of loaded fibers in the bundle. Furthermore, determining the true strength of the fiber bundles is even more difficult since it is practically not possible to check the amount of fibers broken at maximum loading in an observable way. One of the options found in the literature was the detection of rupture signals in bundle displacement ranges [5] or even the determination of the amount of final fibers based on the statistical parameters of the filaments [6]. In general, the mechanical behavior of the fiber largely interferes with the strength of the bundle. However, due to factors such as contact damage, and fiber misalignments, among others, stronger fibers are not necessarily among the last to be broken and this fact also impacts bundle strength.

Another way to investigate the mechanical behavior of the bundle is the load-sharing mechanics between the fibers during individual breakage. Several authors have already studied the load redistribution behavior during fiber bundle failure [7,8,9,10]. In the Global Load Sharing (GLS) models, when one fiber fails, the load is equally divided among all remaining fibers of the bundle [11]. On the other hand, the Local Load Sharing (LLS) models propose that the load carried by the failed fiber is divided only among the neighborhood fibers [12]. For instance, Durham et al. [13] considered a four-neighbor positioning system for the fibers, while Batdorf et al. [14] considered a uniform load sharing among fibers within a determined radius. In addition, a modified local load-sharing model termed non-localized load sharing (NLLS) was introduced recently [15]. This model uses a power law approach for stress distribution as considered by Duxbury and Leath [16] considering that the load carried by the broken fiber is non-uniformly distributed among higher-order neighbors.

The main motivation of this work was to deepen the understanding of the rupture mechanics of ceramic fiber bundles. For this, the effect of the fiber nominal count on the bundle strength was evaluated by means of experiments and simulation. For this, a method was developed to interpret results of tensile tests based on signals of rupture. Through a strain-piloted tensile test, both bundle engineering and true strength were determined for two different types of fibers (3M Nextel 610 and 3M Nextel 720). In parallel, the true strength of bundles with several initial amounts of fiber was also calculated in a force-piloted simulation using three different load-sharing methods. For these simulations, the Weibull parameters of single fibers were used to generate data. By this approach, aspects not experimentally measurable were also explored (e.g., characteristic strength of the last fibers) in order to investigate how the dynamics of fiber breakage during the tests impact the mechanical characteristics of the bundle.

## 2. Materials and Methods

### 2.1. Experimental

#### 2.1.1. Materials

The samples were prepared using commercially available ceramic fiber bundles provided by 3M (St. Paul, MN, USA) [17], whose characteristics are shown in Table 1.

Those fibers were sized by the manufacturer with polyvinyl alcohol (PVA) and other additives such as plasticizers, lubricants, etc., to improve chemical protection and to assist handling. To remove the coating, the fibers were first covered with a thin layer of candle wax. Then, direct fire was applied to the candle wax over the fibers. To perfectly remove both the wax and PVA, the fiber bundle was kept in a horizontal position. With this, significant carbon deposition could be avoided.

#### 2.1.2. Single Fiber Processing and Testing

First, the fiber strength of both 3M Nextel 610 (N610) and 3M Nextel 720 (N720) was tested and fitted with Weibull statistics. Considering this, the single fiber data were examined treated to generate the distribution parameters and compare to the reference data. In addition, single fiber data were also used as simulation input to generate a random population of breaking loads.

The single-filament samples were mounted according to the ASTM D3379-75 [18] standards. This method covers the preparation, mounting, and testing of high modulus single-filament materials (over 21 GPa) for the determination of tensile strength and Young’s modulus at room temperature. According to Wilson [19] and to the standard testing method, the recommended sample length (gauge length) was set to 25 mm for all experiments. The tests were performed using an universal testing machine model DL10000 (EMIC-Instron, São José dos Pinhais, Paraná, Brazil). The machine strain rate for all samples was set to 1 mm min^−1^. Moreover, 33 samples of single N610 fibers and 32 samples of single N720 fibers were tested.

The breaking strength of the filaments was calculated using the ratio between the breaking load and the cross-section of the filament. However, the diameter measurement was not performed for this purpose. Instead, the diameter considered was the average value provided by the manufacturer presented in Table 1 (in this case, 11 μm).

The application of the Weibull distribution to single fibers and fiber bundles was already provided by several authors [7,8,9,13]. The 2-parameter Weibull distribution for the cumulated probability is given by

(1) $$P\left(\sigma \right)=1-\exp\left[-{\left(\frac{\sigma }{\sigma_{0}}\right)}^{m}\right]$$

where σ is the rupture strengh of a sample and P(σ) is the probability of rupture under such stress. $\sigma_{0}$ and m are the Weibul distribution parameters which represent, respectively, the characteristic strength of a given set of tested samples and the Weibull modulus. According to Bernard et al. [20], the probability P(σ) can be estimated by

(2) $$P\left(\sigma_{i}\right)=\frac{i-0.3}{n_{R}+0.4}$$

where $\sigma_{i}$ is the $i^{th}$ rupture strength in increasing order within the *i*th replications of the experiment.

The $\sigma_{0}$ and m parameters for both types of single fibers were then determined using the maximum likelihood method. Respectively, the parameters of the single fibers N610 and N720 are given the symbols $\sigma_{s,610}$ and $\sigma_{s,720}$ for the characteristic strengths and $m_{s,610}$ and $m_{s,720}$ for the Weibull moduli.

#### 2.1.3. True Strength of Fiber Bundles

For the fiber bundles, the modified ASTM test method D4018 [21] was applied using paper tabs from ASTM test method 3379 [18]. In this method, the test specimens require no special gripping mechanisms; therefore, standard rubber faced jaws have been used here.

Fiber bundles were tested with varying fiber bundle size. In these experiments, the engineering and true rupture stresses were investigated. For true strength, a method based on rupture signals is introduced and applied. In the work of R’Mili et al. [5], the acquisition of rupture signals was made by detecting acoustic emissions from fiber rupture. Different from the present work, these signals were organized into classes based on specimen deformation at the time of fiber breakage. This was possible due to the use of an extensometer for the gauge length mentioned in the European Standard EN 1007-5 [22]. In the present work, we instead used a short gauge length as recommended by the ASTM D-4018 standard [21], and no extensometer was used. Moreover, the initial phase of the test commonly presents a fiber alignment phase, which, however, does not configure a pure fiber bundle deformation. Furthermore, in R’Mili et al. [5], the normal distribution needed to be adjusted for the interpretation of the rupture frequency distribution in the deformation classes of the specimen. Lamon et al. [23] used the same approach to investigate the behavior of natural flax fiber.

The fiber bundle samples were assembled according to the same specifications as the single fibers. However, the amount of fiber in each bundle was separated by mass. For both types of fibers, tests were performed on bundles with initial amounts of fibers ranging from 100 to 1500. The tests were performed at a machine strain rate of 1 mm·min^−1^ and were replicated 10–50 times for the sake of statistical significance. Samples that showed detachment, sliding or other adverse behavior were discarded. The number of initial fibers used for each type of fiber and the replications performed for each experimental combination are shown in Table 2.

The engineering strength of a given material is calculated by the ratio of the breaking load to the initial cross-section of the sample. In mathematical terms,

(3) $$\sigma_{eng}=\frac{F_{max}}{A_{0}}$$

where $F_{max}$ is the breaking load and $A_{0}$ is the sample original cross-section, which is given by

(4) $$A_{0}=n_{f}\times A_{1},$$

where $n_{f}$ is the initial amount of fibers and $A_{1}$ is the single fiber cross-section.

However, some materials vary in cross-section due to external loading. On the other hand, in monolithic ceramic materials, such an effect is not observed. In ceramic fiber bundles, however, the cross-sectional area varies during load application as fibers break during the process. When the cross-section variation of a sample is considered to determine its strength, this parameter is called true strength or true rupture strength. However, the exact measurement of the area at the time of rupture is very difficult especially for fiber bundles with a fine diameter. To solve this problem, here, a failure signal is counted each time a negative load variation is recorded during a fiber bundle tensile test, as a negative load variation can be considered as a signal for the stepwise reduction of the fiber count. For the entire experimental design, the load variation between each test step was calculated. Consider this variation as ΔF and assign an S=1 signal to each ΔF<0. In addition, consider $S_{T}$ as the sum of all the signals recorded for a given sample. For each fiber bundle sample, this sum of signals is considered to be proportional to the amount of initial fibers of a given sample. Due to the impossibility of direct measurement, it is not possible to determine how many fibers break at each record. In addition, the acquired signals are classified according to the loading range in which they were recorded. By default, the number of loading range classes is calculated by the square root of the total amount of failure signal records $\sqrt{S_{T}}$. In addition, the load ranges used in each class are uniformly defined with the upper load limit supported by the bundle. Thus, the number of signals for a given loading class i is defined as $S_{i}$. In general, a similar procedure for constructing a histogram of the frequency distribution of fiber rupture signals in classes of load ranges is performed for every tensile test. However, the interpretation of histograms and a possible fit with statistical distributions are not objects of study in this work even though the construction of these diagrams is possible under this context. With this, the applied method was slightly different from that presented by R’Mili et al. [5] in which the acoustic events were classified by strain range classes and fitted to a normal distribution.

Now, for each class of signals, the expression $\frac{S_{i}}{S_{T}}\cdot 100$ results in the percentage of fractured fibers in each loading class. Thus, considering the initial amount of fibers $n_{f}$ for a given test, it is possible to estimate the amount of fibers that fracture in each class of loading using the expression

(5) $$n_{b\left(i\right)}=n_{f}\times \frac{S_{i}}{S_{T}}$$

Finally, the amount of fibers broken at the last moment is the amount of fibers calculated in the last class of the loading range. With this, $n_{last}$ is used to calculate the cross-section at the moment of rupture.

Now we consider the computation of the stress supported by the cross-section of the fiber bundle during the test using $\sigma =\frac{F}{A}$, where A, which is given by $A=n_{f}\times A_{1}$, is the cross-section of the fiber bundle sample at a given moment when F is the applied load at the same moment. Thus, in the $i^{th}$ class of signals, the amount of unbroken fibers is $n_{last}$ and the last load class is $F_{last}$. With this, the bundle true strength can be estimated using

(6) $$\sigma_{true}=\frac{F_{last}}{n_{last}\times A_{1}}$$

**Figure 1:**
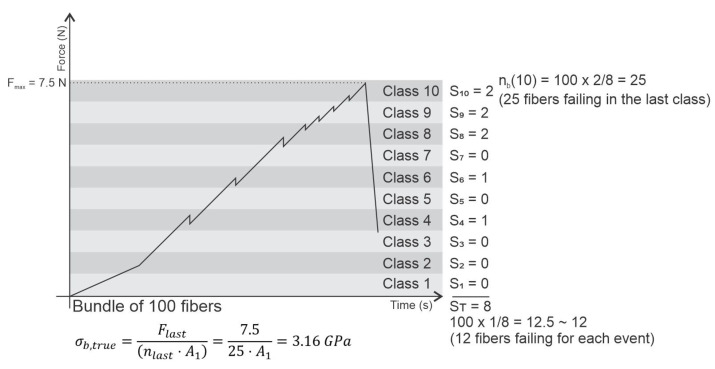
Schematic representation of the procedure to determine the true strength of a fiber bundle sample.

A schematic representation of the procedure for obtaining the true strength of a sample is shown in Figure 1.

As for the filaments, the synthesis of the results of $\sigma_{true}$ was done using Weibull statistics. Thus, for each type of fiber and for each initial quantity of fibers in the bundle, the characteristic true strength and the Weibull modulus were determined. For such parameters, the symbols $\sigma_{b,true}$ and $m_{b}$ are assigned

Regarding $n_{f}$, this parameter was considered to be equal to the nominal quantity described in Table 2 in this method. It is known that, in the work of R’Mili et al. [1], efforts are directed at determining the exact amount of fibers loaded at the beginning of the test. It is a method based on strain-load curves, where the slope of the linear region of the graph together with the filament tensile modulus determines the amount of fibers loaded. This amount is given as

(7) $$n_{in}=\frac{S_{o}}{A_{1}\;E_{f}}\times n_{f}$$

where $S_{o}$ is the initial slope, $A_{1}$ is the fiber cross-section, and $E_{f}$ the filament modulus.

In addition, Lamon et al. [6] defined the tow strength as

(8) $$\sigma_{F}=\frac{F_{max}}{A_{o}\left(1-\gamma \right)\left(1-\alpha \right)}$$

where γ is the fraction of fibers broken prior to testing, given as

(9) $$\gamma =1-\frac{n_{in}}{n_{f}}$$

and α is the fraction of broken fibers at each instant; its critical value is given as

(10) $$\alpha_{c}=\frac{n_{C}}{n_{f}},$$

where $n_{C}$ is the number of fibers broken at maximum load. In addition, the author also mentions that $\alpha_{c}$ can be accurately estimated through

(11) $$\alpha_{c}=1-\exp\left(-\frac{1}{m}\right)$$

where m is the Weibull module of the samples.

On the other hand, in this work, it is defined as the proportion of remaining fibers given by

(12) $$R_{p}=\frac{n_{last}}{n_{f}}.$$

Unlike the work by Lamon et al., this proportion does not necessarily represent the proportion of the strongest fibers among the bundle, but it represents the proportion of fibers surviving at the final moment of rupture.

Another approach is still possible for the determination of the fraction of the last fibers in the bundle. Considering the fibers being purely elastic with uniform Young’s modulus, it would be possible to calculate

(13) $$\alpha_{alt}=\frac{\Delta F}{F_{max}+\Delta F},$$

where ΔF is the difference between the theoretical maximum load of the sample and the maximum load $F_{max}$ recorded in the experiment. The maximum theoretical load Fteo is obtained by means of the linear elastic relation

(14) $$F_{teo}=k\;x_{rup},$$

the value of k being determined experimentally for each replication of the experiment. $x_{rup}$ is the abscissa value at which $F_{max}$ is observed. Thus, even though an extensometer was not used, the value of $x_{rup}$ could be calculated using a load versus time graph. In addition, the $\alpha_{alt}$ value already considers the unloaded fibers prior to the start of the test since the k value reveals the slope of the theoretical curve, which, in itself, is dependent on the number of fibers initially loaded. Finally, for this approach, the value $\left(1-\alpha_{alt}\right)$ also represents the fraction of the initial cross-section present at the moment of rupture.

### 2.2. Simulation

In the simulations, a numerical method was performed using three load-sharing models known in the literature (GLS, LLS, and NLLS). As input data, the statistical parameters obtained from the N610 and N720 filaments tested were used. Enhancing previous simulations [15], a self-growing hexagonal fiber placement setup was developed to represent the circular cross-section arrangement within a bundle. This approach was based on the triangular lattice geometry of Derda [24]. This option avoided some problems related to the n by n square matrix approach and allowed the simulation to be executed for almost any nominal count of virtual fibers [4,15].

The virtual fiber bundle is considered a group of $n_{f}$ breaking load values randomly generated using the Weibull distribution of the strength of single fibers. With this, $n_{f}$ values of P(σ) were randomly generated where 0<P(σ)<1. Thereby, $n_{f}$ is the amount of fibers in the virtual bundle. With this, the $n_{f}$ breaking loads F needed for the simulation are generated by using

(15) $$F=\sigma \;A_{1}$$

For the load-sharing models with neighborhood configurations, a new setup of fiber arrangement was developed in order to overcome the limitations of the square or rectangular cross-section of virtual fiber bundles used previously [4,24,25,26]. Here, a hexagonal arrangement system was developed aiming to avoid the irregular stress concentration on the edges of the square or rectangular arrangement system.

**Figure 2:**
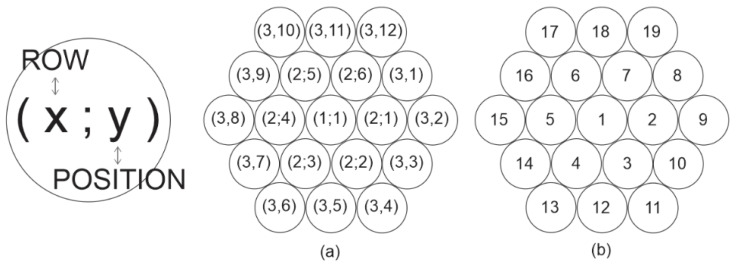
Virtual bundle position matrix representation for $n_{f}=19$: (**a**) Matrix indexes and (**b**) virtual fiber ID.

For the analysis, the positioning system must be consistent and self-organized in order to map and find the neighborhood easily. Thereby, matrix cell indexes were given to the fibers of the bundle. These indexes are classified in rows (x value) and position (y values) as presented in Figure 2. Thereby, identity numbers are given to each fiber of the virtual bundle to ease the simulation process.

The simulation aims to test virtual bundles until complete failure. This is done by performing a stochastic procedure by increasing the virtual load, comparing with the virtual bundle data, and applying load-sharing conditions.

For example, let L be the applied load and ΔL the load increment for every step of the simulation which is set manually. Thereby, high ΔL values provide large amounts of virtual fiber failures per step, which leads to a low-resolution simulation. On the other hand, low ΔL values tend to require a high processing effort. Thereby, a ΔL suitable for the single fiber tensile tests was used in the simulations, e.g., ΔL=4,5 N for N610 and ΔL=1 N for N720.

In addition, let $\bar{A_{L}}$, calculated by $\bar{A_{L}}=L\cdot \bar{k}$, be the applied load and $\bar{k}$ the load sharing factor matrix. It is important to note that the $\bar{A_{L}}$ and $\bar{k}$ matrices have the same structure as a virtual bundle $\bar{B}$ constituted of $n_{f}$ fibers. With this, it is possible to directly compare all $\bar{A_{L}}\left(i,j\right)$ with $\bar{B}\left(i,j\right)$. The comparison between these matrices allows the code to analyze which fibers are broken. This is done by $\bar{A_{L}}\left(i,j\right)>\bar{B}\left(i,j\right)$. The load factor matrix $\bar{k}$ represents how much load is applied to each fiber of the virtual bundle. At the beginning, all $\bar{k}\left(i,j\right)$ values are set to one, which means that every element receives 100% of the load L applied to it. Thereby, the factor for the broken fibers are set to zero ($\bar{k}{\left(i,j\right)}_{broken}\to 0$). At this point, the load factor for the surviving fibers of the bundle will receive values above one. The computation of this value depends entirely on the load-sharing model implemented in the simulation.

**Figure 3:**
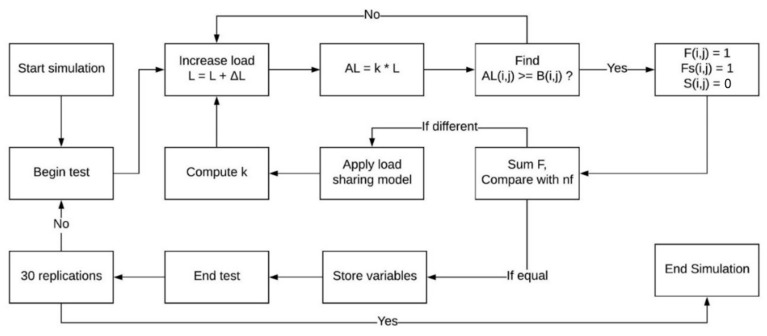
Simulation Process Flow Chart.

Let $\bar{F}$ be the failure matrix, $\bar{F_{s}}$ the step-failure matrix, and $\bar{S}$ the survivors’ matrix. All three matrices have the same structure as $\bar{B}$. The central values of the $\bar{F}$ and $\bar{F_{s}}$ matrices are set to zero, which represents no initial broken fiber. When a virtual fiber breakage is computed at (i,j), the values of $\bar{F}\left(i,j\right)$ and $\bar{F_{s}}\left(i,j\right)$ equal 1. With this, it is possible to map and to count the amount of broken fibers of the virtual bundle. When the sum of all terms of $\bar{F}$ becomes equal to $n_{f}$, the simulation finishes. This sum is considered as $n_{fail}$. In the simulation, $\bar{F_{s}}$ only stores the fractured fiber for a specific step. The values of this matrix are used to calculate the load-sharing factors of $\bar{k}$. After every step, the values of $\bar{F_{s}}$ are set again to zero. The survivors’ matrix $\bar{S}$ values are initially set to one, as those fibers are still unbroken. When a fracture is detected on fiber (i,j), the value $\bar{S}\left(i,j\right)$ is zero, e.g.,$\;\bar{S}{\left(i,j\right)}_{broken}\to 0$. The survivors’ matrix allows the calculation of the load-sharing factor. This is done by verifying in the $\bar{S}$ matrix if a fiber which experiences some shared load is still unbroken. The main scheme of the simulation procedure is presented in Figure 3.

At the end of the simulation, the bundle’s simulated true strength is calculated using the expression

(16) $$\sigma_{sim,true}=\frac{F_{last}^{*}}{n_{last}^{*}\times A_{1}}$$

where $F_{last}^{*}$ is the last load carried by the bundle and $n_{last}^{*}$ is the final amount of fibers.

The simulated characteristic strength of the last fibers within the bundle, referred to as $\sigma_{sim,LF}$, is also determined. Different from the experiments, it is possible to verify the breaking strengths of the last fibers within the virtual bundle. With this, for every replication of an $n_{f}$ fiber bundle, the characteristic strength of the remaining fibers is determined. Likewise, the Weibull characteristic strength is determined for the population of 30 replications of $n_{f}$ fiber bundles to synthetize and present the data. With this, $\sigma_{sim,LF}$ is obtained. In addition, the $R_{P}$ ratio is also determined for the simulated data for comparison with experimental data. This simulated ratio is given by

(17) $$R_{sim,P}=\frac{n_{last}^{*}}{n_{f}}$$

#### 2.2.1. Global (or Equal) Load Sharing (GLS)

In this model, the load carried by single broken fibers is equally divided among all remaining fibers of the bundle [11]. This model does not require any neighborhood identification. Thereby, the load-sharing factor $\bar{k}$ depends only on the surviving fiber number $n_{s}$ given by

(18) $$n_{s}=n_{f}-n_{fail}$$

In addition, once a virtual fiber $\bar{B}\left(i,j\right)$ fails, its factor $\bar{k}\left(i,j\right)$ is divided by $n_{s}$. This value can be called δk and is computed by

(19) $$\delta k=\frac{\bar{k}\left(i,j\right)\;}{n_{s}}$$

For the global load-sharing rule, the δk value is added directly to the load-sharing factor of all the remaining $n_{s}$ fibers. At the same time, the load-sharing factor of the failed fiber automatically receives a value of zero.

#### 2.2.2. Local Load Sharing (LLS)

In this model, the load carried by the failed fiber is shared with the local neighbor fibers [12]. The local neighborhood is defined by the surviving fibers in contact with the failed fiber. Thereby, the number of direct neighbors of a single fiber varies between 1 and 6 in the hexagonal arrangement. Once a broken fiber is detected, the neighborhood mapping code detects which fibers are part of the hexagonal neighborhood. The mapping procedure also considers the fibers which are at the boundaries of the bundle, which do not have the same fiber neighborhood. Thereby, the number of surviving fibers of the local neighborhood is $n_{sl}$ and the shared factor of this model is given as

(20) $$\delta k=\frac{\bar{k}\left(i,j\right)}{n_{sl}}.$$

After the detection of the local neighborhood, the δk value is added directly to the load-sharing factor of the surviving neighbors. Here, it is important to mention that the sum of the elements of the $\bar{k}$ matrix must be equal to $n_{f}$.

#### 2.2.3. Global non-Localized Load Sharing (NLLS)

As already mentioned, this model is related to the LLS model, but the factor accumulated on the surviving local neighbors is spread to a further neighborhood [15]. Similar to the Duxbury and Leath model [16], load distribution based on a power law is applied to higher-order neighborhoods in this model. However, the procedure applied in the model of this work is numeric. First, the $\bar{k}\left(i,j\right)$ factor of the broken fiber is directly shared with the surviving local neighbors as in the case of the LLS rule. After that, a neighbor-mapping routine is used to identify and count the remaining unbroken neighboring fibers of the bundle. For these fibers, the neighbor mapping process code calculates the average value of the of the unbroken neighbor fibers’ k factor together with its own k value. Then, these same fibers receive this average k factor value. Mathematically, for a unbroken fiber which will receive the new factor $k_{n}$, $n_{nsf}$ is the amount of neighboring unbroken fibers of one specific element (also counting the given fiber itself) and $k_{nsf}$ is the sum of the k factor of the $n_{nsf}$ virtual fibers. Thereby, $k_{n}$ is defined as

(21) $$k_{n}=\frac{k_{nsf}}{n_{nsf}},$$

where $k_{n}$ is the new k factor of this specific element of the nearby fiber failure.

With this, unbroken virtual fibers at a distance further than one row from the broken fiber receive an extra factor, which is the scattering of the broken fiber shared load. Again, the mapping procedure also considers the fibers which are at the boundaries of the bundle, which do not have the same fiber neighborhood.

## 3. Results and Discussion

### 3.1. Strength of N610 and N720 Single Fibers

**Figure 4:**
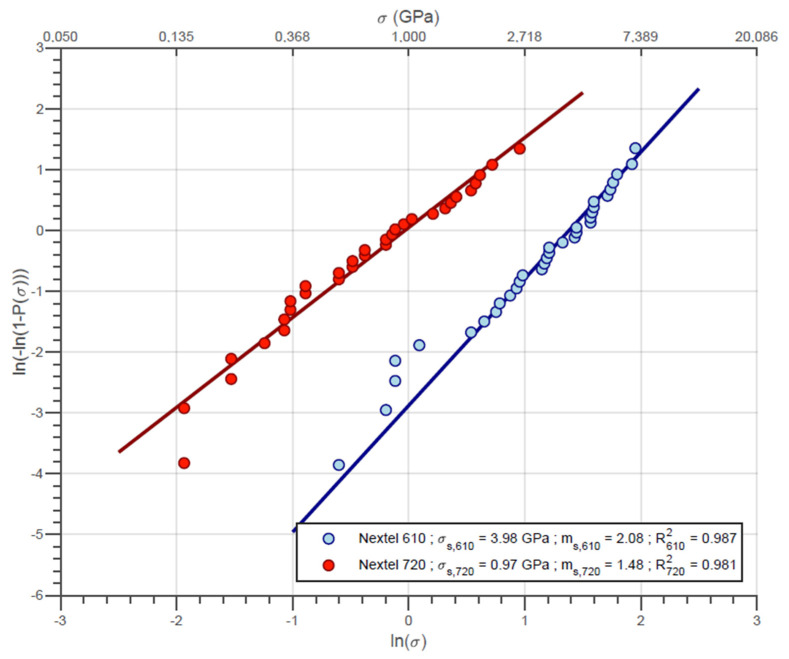
Weibull plot for N610 and N720 single fibers.

The Weibull plot for the single fiber samples is presented in Figure 4. N610 fibers are made of 99% pure α-alumina which provides a very strong atomic bonded material. Using the short gauge length (≈25 mm) and the medium fiber diameter of 11 μm, the strength of the N610 fiber is 3.98 GPa with minimum and maximum being 0.48 GPa and 6.71 GPa, respectively. The respective characteristic strength of the N720, calculated using the same diameter, is approximately 0.97 GPa, with minimum and maximum values of 0.14 GPa and 2.40 GPa. The low strength of N720 is mainly caused by globular grains of mullite with 0.5 μm diameter, e.g., five times larger than the alumina grains in N610 fibers. In addition, the low fracture toughness of mullite limits the strength of these fibers when compared to alumina [19]. It is important to note here that the strain rate during the test is considerably higher than the one used in the work of Wilson [27], e.g., 1 mm·min^−1^ instead of 0.02 mm·min^−1^. On the other hand, the Weibull modules obtained for the filament samples are significantly smaller than the values published by Wilson. This difference is possibly associated with the difficulty in preparing and handling the samples. Moreover, it is also not impossible (also unlikely) that the adopted de-sizing process affected the fibers.

### 3.2. N610 and N720 Fiber Bundles

#### 3.2.1. Engineering Strength of N610 and N720 Fiber Bundles

**Figure 5:**
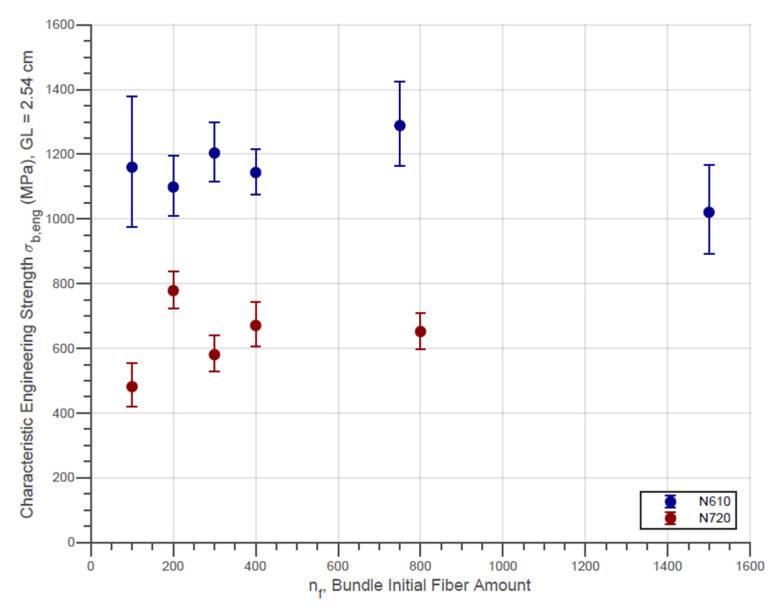
Characteristic Engineering Strength of N610 and N720 bundles with different initial fiber amounts.

Fiber bundles tests were performed with 100, 200, 300, 400, 750, and 1500 fibers for N610 and with 100, 200, 300, 400, and 800 fibers for N720. The Weibull characteristic engineering strength for the N610 and N720 bundles with different initial fiber amount is presented in Figure 5. The engineering strength for every test replication, however, was obtained using Equation (3). The error bars represent the 95% confidence intervals for the parameter estimates in Figure 5.

In this figure, it is important to emphasize that the results obtained are for samples with a 2.54 cm gauge length (GL). In the previous work of Neckel, et al. [15], the studies were focused on determining the engineering strength of Nextel 610 fiber bundles with a 10 cm gauge length for comparison with the simulations developed at the same time. A comparison can be made using the engineering strength correction as a function of the length as presented by Wilson [27]. The correction equation is given by

(22) $$\frac{\sigma_{1}}{\sigma_{2}}={\left(\frac{V_{2}}{V_{1}}\right)}^{\frac{1}{m}}$$

where $\sigma_{1}$ and $\sigma_{2}$ are the Weibull characteristic engineering strengths of samples with respective volume given by $V_{1}$ and $V_{2}$, and m is the sample set of the Weibull modulus.

Since the cross-section area is invariable for the engineering strength calculation, it can be simplified from the expression, which gives

(23) $$\frac{\sigma_{1}}{\sigma_{2}}={\left(\frac{L_{2}}{L_{1}}\right)}^{\frac{1}{m}}$$

where $L_{1}$ and $L_{2}$ are the gauge lengths of the samples.

In previous work [15], the N610 engineering strength for the 10 cm gauge length as-received samples ($n_{f}=750$) was $\sigma_{1}=919.72\;\mathrm{MPa}$ with m=6.17. Using $L_{2}=2.54\;\mathrm{cm}$, which is the gauge length used for the experiments in the present research, we obtained $\sigma_{2}=1151.4\;\mathrm{MPa}$. Being aware of the possible statistical variations and random errors associated with the experiments, it is considered that the results presented in Figure 5 are adequately similar to the result obtained through the correction equation.

In addition, Figure 5 also shows that the engineering strength of the different bundles of the same fiber type have approximately constant values, except for random errors. This fact corroborates the literature since engineering strength is not affected by the cross-section of the sample. Moreover, the experimental parameters such as maximal force, engineering strength, and Weibull modulus for the fiber bundles are presented in Table 3. The Weibull moduli for the N610 and N720 fiber bundles samples are, however, below the values in the literature [15,27]. As with simple fibers, this fact is associated with handling difficulties that possibly cause a wide range of engineering stress values for different bundles with the same initial amount of fibers.

In addition, as previously mentioned, it is not impossible (also unlikely) that the adopted de-sizing process affected the fibers. However, the Weibull modulus presented by the N610 bundles with 750 fibers (as-received) is not significantly different from that obtained in the previous work.

#### 3.2.2. True Strength of N610 and N720 Fiber Bundles

At the beginning of every test, a specific stretching effect is always observed. During this period, non-loaded fibers are stretched and aligned in order to also carry some load. This fact causes the lack of linearity at the beginning of the test. In addition, in some samples, a variation of slope is also noticed. These variations are also associated with the initial loading of unstressed fibers at the beginning of the test. In addition, because an extensometer was not used, the deformation record presented by the machine does not represent the strain of the material in a reliable way. Moreover, it is recalled that the objective was to map the amount of rupture signals for different loading ranges and, for this reason, the use of such equipment was discarded.

**Figure 6:**
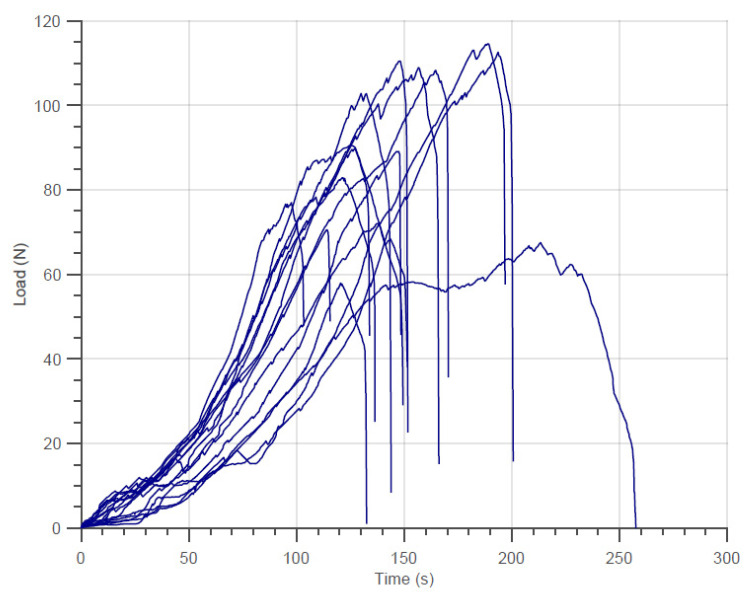
Examples of Tensile Tests on N610 with 750 fibers bundles.

During tensile testing of these bundles, the weakest fibers break first as expected. At every fiber breakage the supported load decreases a little, but with enough number of surviving fibers, it normally increases again. An example of the loading versus time diagrams is presented in Figure 6. In this diagram, only the results of the tests with the N610 samples with 750 fibers are presented. The other diagrams (other samples of N610 and N720), despite showing similar details, although with different maximum loads, were not presented to avoid overloading the document. In addition, the diagram shows the variation of the force applied against the test time due to the facts mentioned above.

The negative variations in the load supported by the bundle as shown in Figure 6 are associated with the rupture of one fiber or a group of fibers in the bundle. As mentioned in the previous section, the breaking load registered in every run is used to estimate the engineering strength of the bundle (Figure 5), and the negative variations registered during the tensile tests are used to estimate the true strength. Here, it is important to mention that the registration of the rupture signals and the procedure described for obtaining the final amount of fibers in the bundle were performed individually for each replication of the experiments. The average rupture signal amount and its standard deviation for the different fiber types and initial amount of fibers are presented in Table 4.

**Figure 7:**
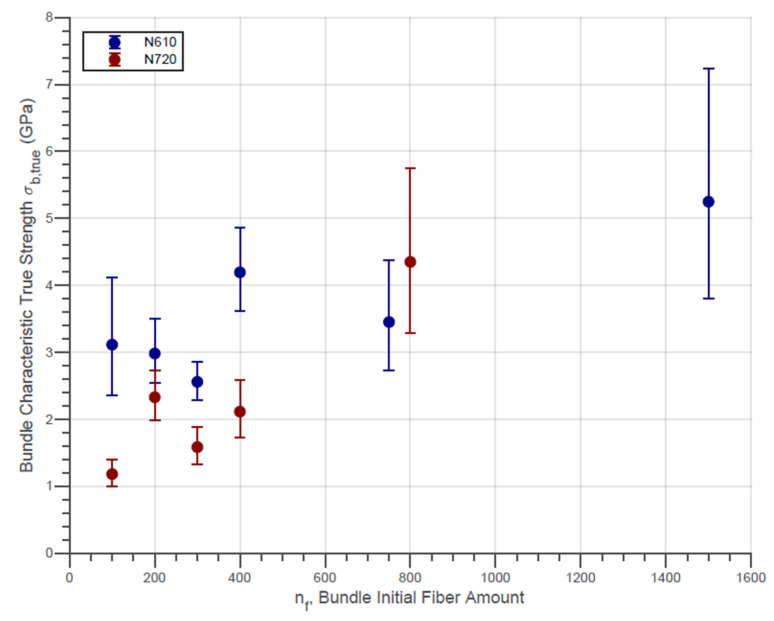
Weibull Characteristic True Strength and Confidence Interval of Fiber Bundles with $n_{f}$ Fibers.

In addition, for each type of fiber and for each group of samples of different amounts of initial fibers, the characteristic Weibull parameters were determined. The evolution of the characteristic true strength between groups of samples with different initial amounts of fibers is presented in Figure 7. In addition, the error bars represent the 95% confidence intervals for the parameter estimates in the same figure.

The data confirm the absence of a simple linear dependency of the bundle strength on the fiber count. In general, the N610 alumina fiber bundles exhibit only a moderate strength improvement with increasing fiber count. On the other hand, the strength of the N720 bundles shows a low but continuous improvement with increasing fiber counts. For low $n_{f}$ values, however, the diagram shows that N610 fibers have an opposite trend. The authors believe that this result is a statistical fluctuation since the central parameter is within the confidence interval of the others ($n_{f}$ = 100–300). It is also remarkable from the same figure that the values found for the $\sigma_{b,true}$ of both fibers are greater than the strength of the filaments themselves. This can be noticed since only a fraction of fibers much less than the original amount bears the maximum load. Even though the maximum load can be considered linearly dependent on the $n_{f}$ (as shown in Table 3), possibly the fraction of fibers surviving at the moment of rupture varies with $n_{f}$ due to factors related to the local load distribution.

**Figure 8:**
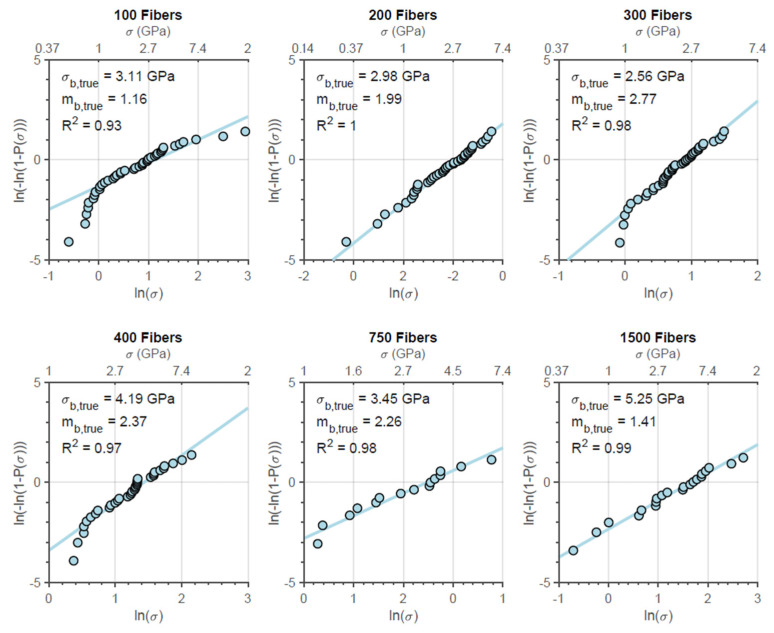
Weibull plots for N610 fiber bundles with different initial amounts of fibers.

**Figure 9:**
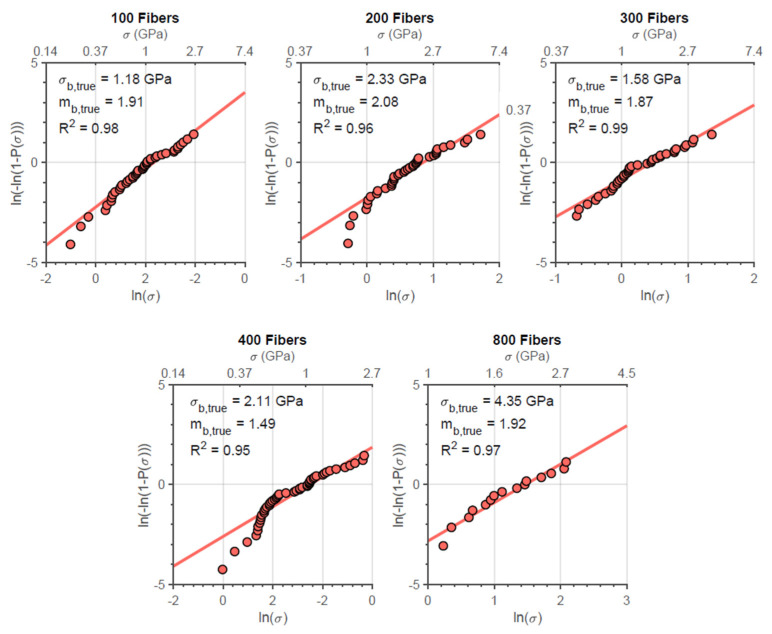
Weibull plots for N720 fiber bundles with different initial amounts of fibers.

The Weibull plot for the true strength of both fiber bundle types for the different initial amounts of fiber tested is presented in Figure 8 and Figure 9. In addition, it is possible to observe through the correlation coefficient presented in the graphs that the true strengths of the fiber bundles are significantly distributed according to the Weibull statistics as well as the engineering strength, which was verified by Neckel et al., for N610 [15] and by Dassios et al. for N720 [7].

In the present procedure of obtaining $\sigma_{true}$, the amount of broken filaments at each ΔF<0 is considered constant. However, it is known that for higher loads, in theory, more filaments break with each record of the rupture signal. Thus, it is possible that the model presented overestimates the true strength of the fiber bundles since the amount of fibers broken with higher loads may be greater than that calculated using the procedure presented.

The Weibull characteristic tow strength was calculated according to Lamon et al. [6], Table 5. Such values were obtained through the expression given by Equation (7) using $m_{s,610}$ to $\alpha_{610}$ for N610 and $m_{s,720}$ to $\alpha_{720}$ for N720. These values were calculated considering the Weibull modulus data obtained in this work and with the data available in the literature. Wilson [27] presents average values of 11.4 for the N610 filament modulus and 7.6 for the N720 filaments. For these values, it is notable that the $\alpha_{c}$ values presented are different since the Weibull modulus obtained in this work is significantly smaller than that presented in the literature. As previously mentioned, the difference in this parameter is associated with the difficulty in handling and separating single fibers during sample preparation. Naturally, this results in a lower strength for the strength obtained considering Wilson’s Weibull modulus since more fibers would be present at the time of the final break.

Furthermore, the γ values were determined using Equation (9) presented in the previous section and were used together with the rupture signal procedure described in this work to determine the tow strength of the bundles in every replication of the experiments. As previously mentioned, γ is the fraction of fibers broken prior to testing. The symbol $\sigma_{true}^{*}$ was used to represent this parameter. These values are also shown in Table 5 together with the average γ value for each experimental combination. Different from the $\sigma_{true}$ values, these values are not significantly variable with $n_{f}$ and are possibly overestimated. Moreover, a reduction in the γ value is also noticed for the different initial amount of fibers in the bundles. For low $n_{f}$ (100, for example), it is believed that the difficulty encountered in separating the amount of fibers by means of their mass has affected these results. On the other hand, for large bundles (1500 and 800 in N610 and N720, respectively), the low γ value is possibly associated with the fact that bundles of smaller amounts of fibers (750 and 400 for N610 and N720, respectively) were merged together. Thus, it eliminates the possibility that this reduction is a phenomenon associated with the amount of fibers in the bundle.

In terms of obtaining the strength of the bundle at maximum load, it is natural that the values obtained for $\sigma_{F}$ are higher compared to the values of $\sigma_{eng}$ This is associated with the fact that, for the same maximum load, a smaller cross-section is considered in Equation (7). On the other hand, the values calculated by Equation (7) are significantly lower than those presented in Figure 7. For the same maximum load, this means that the fraction of fibers remaining at that time is smaller than that proposed by Equation (7). This fact demonstrates that the assumption that some stronger fibers break before the maximum load is plausible. Still, it also points out that possibly the fraction of last remaining fibers, $R_{p}$, is variable with $n_{f}$ since there is a significant increase in the values of $\sigma_{true}$ presented in Figure 8.

As it is possible to observe, the results obtained for tow strength are directly linked to the determination of the real fraction of the cross-section at the moment of the final rupture. As already shown, different models were used for this purpose. A comparison between the results obtained through the different approaches is shown in Table 6. Lamon et al. [6] provide an interesting result in terms of stress as the authors considers the amount of broken fibers at maximum loading and disregards non loaded fibers before the start of the test. However, fibers break in the interval between reaching the linear load and the maximum load due to several factors and their absence is not considered in the calculation. In relation to the final cross-section of the sample, the term (1−γ)⋅(1−α) of Equation (8) represents the fraction of the initial cross-section that it is intact at the time of the break. $R_{p}$, however, presents the fraction of unbroken fibers at the moment of the final rupture by means of the procedure described on this work. On the other hand, the $R_{p}^{*}$ value represents the same value, but considering γ prior to the calculation (same as $\sigma_{true}^{*}$).

First, it is important to note that the value of $R_{p}^{*}$ for N610 $n_{f}$ = 1500 is higher than its $R_{p}$. It is also necessary to verify that γ was calculated individually for all replications of the experiment and considered prior to the processing of rupture signals. The value shown in the table, however, is the average value found for the parameter. It is also important to note that $R_{p}^{*}$, unlike $R_{p}$, presents approximately constant results for the different $n_{f}\;\mathrm{values}$. However, it is possible to understand that, for $R_{p}^{*}$, the reduction of $R_{p}$ is compensated for by the variation of gamma with $n_{f}$. In any case, the values of $R_{p}$ and $R_{p}^{*}$ are lower than the values presented through the theory (both $\left(1-\alpha_{c}\right)\left(1-\gamma \right)$ for Weibull modulus from this work and from the literature). For such verifications, two explanations are plausible: the model of the present work overestimates the amount of fibers broken prior to the moment of rupture and/or the theoretical model overestimates the amount of fibers present in the final break because it does not consider that more fibers may break before that moment.

In addition, the values of $\left(1-\alpha_{alt}\right)$ obtained by Equation (13) are also shown in Table 6. These values are also apparently constant with $n_{f}$. On the other hand, this parameter is also shown to be inferior to the fraction of the cross-section calculated using the theoretical $\alpha_{c}$ obtained in this work and through the literature. This fact reinforces the assumption that stronger fibers will break prior to the maximum load. On the other hand, the non-uniformity of the loading curves (as shown in Figure 6) may have resulted in very scattered γ and $\alpha_{alt}$ values since they were obtained for each replication of the experiments. In terms of true strength, the values used $\left(1-\alpha_{alt}\right)$ for the determination of the cross-section at the moment of rupture would also be constant, but naturally higher than the theoretical tow strength presented in Table 5. Finally, the plausibility that the true strength of the fiber bundles may be greater than the filament strength are presented in Table 5 and Table 6. Thus, further analysis of the methods for estimating the amount of broken fibers during the tests is necessary.

An additional parameter that could be analyzed would be the characteristic strength of the last fibers of the bundle $\sigma_{LF}$. Through the experimental design performed, this parameter would indicate if the last fibers to break last have different strengths for bundles with more or less filaments. Considering the statistical distribution of fiber strength, it is expected that such a parameter would increase for bundles in which $R_{p}$ undergoes a reduction since a smaller range of greater strengths would be selected. On the other hand, this parameter would prove to be constant if the fraction of fibers at the moment of rupture did not vary with $n_{f}$, as suggested by the other models considered. In theory, the proportion of fibers remaining at the last moment of the test is the strongest among the population of $n_{f}$ fibers. However, this explanation is only plausible if a uniform load redistribution throughout the test (GLS) is considered. On the other hand, if a local type of load redistribution (LLS) is considered in the event of fiber breaks during the test, it is possible that, at the time of the final break, the remaining fibers are not necessarily the strongest among the population of $n_{f}$ fibers. With this, lower $\sigma_{LF}$ values would be expected. However, it would not be possible to experimentally determine the characteristic strength of the last fibers within the bundle since it is not possible to separate the last fibers for every test. In addition, since the experiments were developed in a strain-piloted test, the negative variations in loading represent the loading of some unstretched fibers in sequence to the breaking of those overloaded. In this way, the hypotheses of the influence of the load-sharing model are more plausible for force-piloted tests. Moreover, it is possible to obtain a comparison for discussion through the simulations since, different from the experiments, it is possible to acquire and analyze the strength of the fibers to be broken off in the last step of the simulation.

### 3.3. Simulation

With the N610 and N720 single fiber data generated from the experiments (Figure 6), 30 virtual bundles from 100 to 1500 fibers were generated using Equation (14) and Equation (15). With this, bundles were submitted to the simulation until final failure. As mentioned, the computational procedure was carried for the different load-sharing models presented in the previous section using the same virtual set of bundles.

Obviously, a negative variation of the external load during fiber breakage cannot be simulated. However, the simulations provide the amount of unbroken fibers at each step. With this, the true strength of the bundles together with the true strength of fibers within the bundle can be modeled.

**Figure 10:**
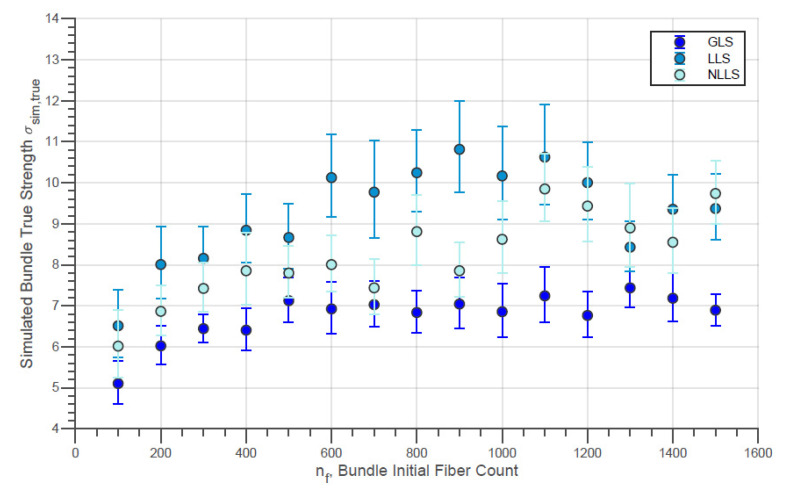
Simulated Characteristic Bundle True Strength against Initial Fiber Count for N610 Fiber Bundles using different load-sharing models.

**Figure 11:**
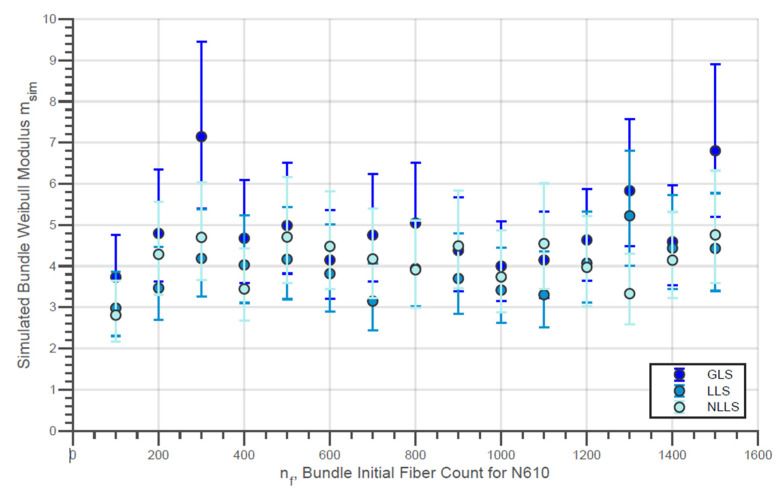
Simulated Weibull modulus for the true strength of fiber bundles of N610 fibers with different load-sharing models.

**Figure 12:**
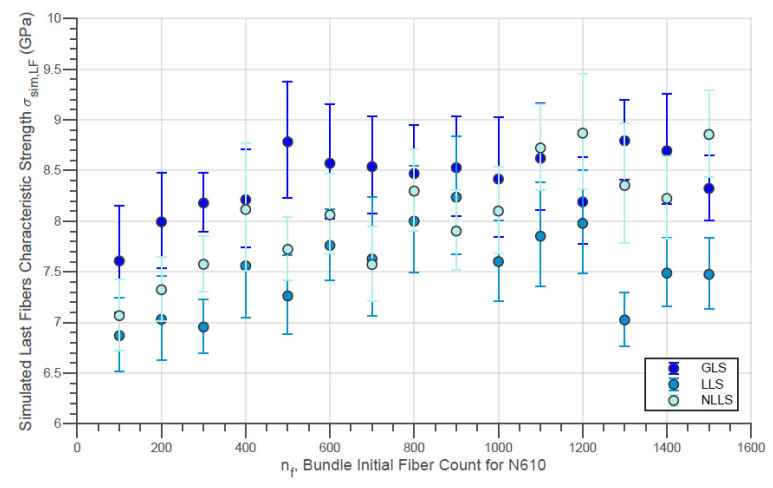
Simulated Characteristic Strength of Last Fibers Within the Bundle against Initial Fiber Count for N610 Fiber Bundles using different load-sharing models.

The evolution of the simulated Weibull characteristic true strength $\sigma_{sim,true}$ of the fiber bundles with different initial fiber counts with the three different load-sharing models is presented in Figure 10. In addition, the evolution of the simulated Weibull moduli $m_{sim,true}$ for the same conditions is shown in Figure 11. Moreover, the simulated characteristic strength of the last fibers within bundle $\sigma_{sim,LF}$ is presented by Figure 12. In addition, the error bars presented in these figures represent the 95% confidence interval for the parameter estimate.

A small increase in true strength for the N610 bundles in the range of low fiber counts can be noted in Figure 10. However, at higher fiber counts, e.g., above 600 fibers, the bundle strength remains almost constant. Thereby, it is interesting to note that the LLS model indicates a reduction of the true strength for larger counts ($n_{f}>1000$). On the other hand, the simulated Weibull modulus for all simulated $n_{f}$ values presented in Figure 11 is practically constant for all load-sharing models. However, it is interesting to note that, in general, the GLS model returns values higher than the values of the LLS. In fact, there are data in which the simulated module in the GLS is up to two units higher than the other models (e.g., $n_{f}$ = 300and $n_{f}$ = 1500). Such verification is plausible since, in the GLS model, it is expected that the order of breaking of the fibers will follow the increasing order of the breaking strength of the filaments present in the bundle. On the other hand, a wider distribution of maximum loads (and true strength) is expected since once in the LLS and NLLS, stronger fibers can receive an overload and break before the individual external load reaches the load of rupture of these fibers. In addition, in LLS and NLLS, the formation of a broken fiber cluster is possible in the same way that regions not affected by the load distribution may exist. This allows the breaking load to be greater for the LLS compared to the GLS. This then results in true strength values that are also higher for LLS compared to GLS (considering that $R_{p,sim}$ is not significantly affected by the load-sharing model)

In addition, the characteristic strengths of the last fibers presented by Figure 12 are considerably lower in the LLS model compared to the GLS for all $n_{f}$ values. This fact reveals that, when considering a local load-sharing model, there is a possibility that stronger fibers will break before the final breakage. Likewise, it is also possible that weaker fibers, which were not affected by localized sharing, are present in the final break. Considering this, the strength distribution of the last fibers tends to have a smaller locality parameter compared to the results from global load-sharing models.

**Figure 13:**
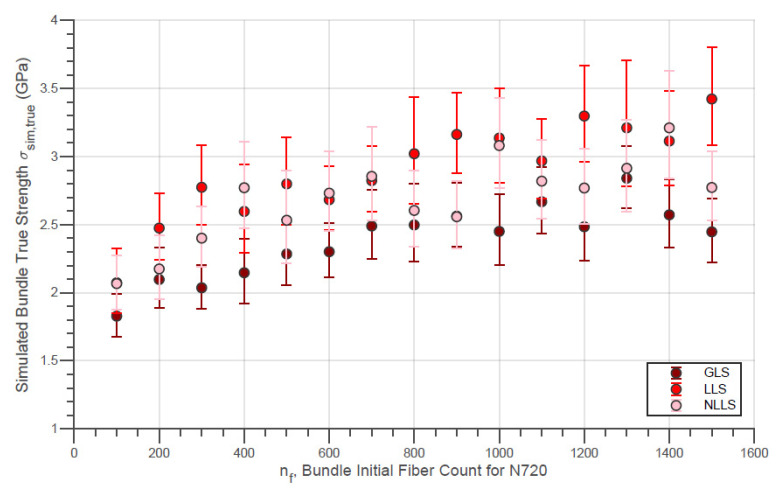
Simulated Characteristic Bundle True Strength against Initial Fiber Count for 720 Fiber Bundles using different load-sharing models.

**Figure 14:**
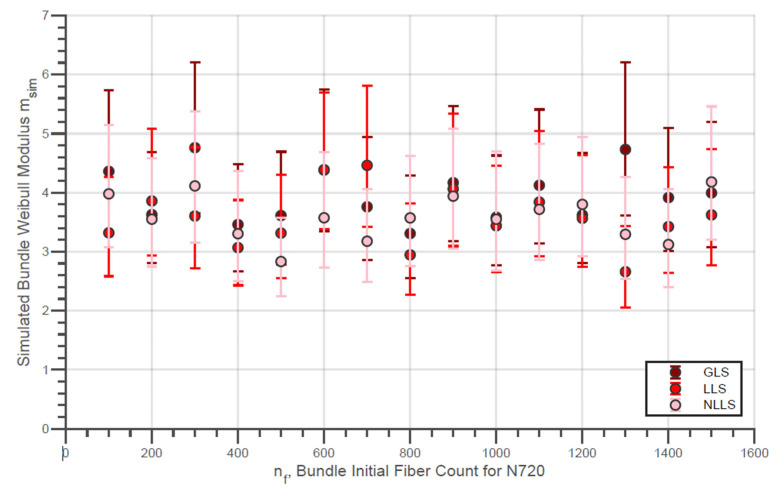
Simulated Weibull modulus for the true strength of fiber bundles of N720 fibers with different load-sharing models.

**Figure 15:**
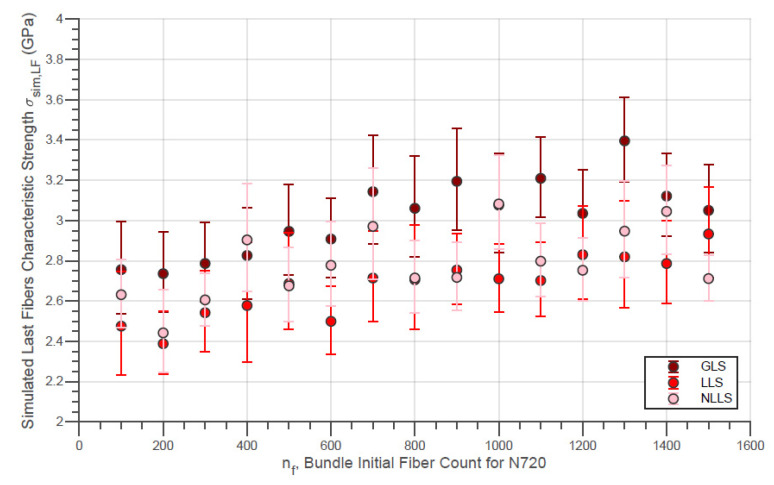
Simulated Characteristic Strength of the Last Fibers Within the Bundle against Initial Fiber Count for N610 Fiber Bundles using different load-sharing models.

In the same sequence, the same simulation results for the virtual N720 fiber bundles are presented by Figure 13, Figure 14 and Figure 15. For N720 fibers, the true strength (Figure 13) exhibits a noticeable increment for the whole range of simulated $n_{f}$ for all load-sharing models. Thereby, the load-sharing models analyzed resulted in overlapping distribution functions for both fiber types so that at present a deeper interpretation is not possible. In addition, practically the same observations made for the results from the simulations with the N610 bundles are possible for the figures related to the N720 fiber bundles.

It is possible to observe that the Weibull modulus of the filaments, as an input parameter of the simulations, has an influence on the results presented. Initially, there is a slight difference between the Weibull module presented by the N610 bundles and the N720. This parameter is notably lower for the N720 compared to N610. The average of the Weibull modulus (considered constant for different $n_{f}$) for the different load-sharing models for the two types of fibers is presented in Table 7.

**Figure 16:**
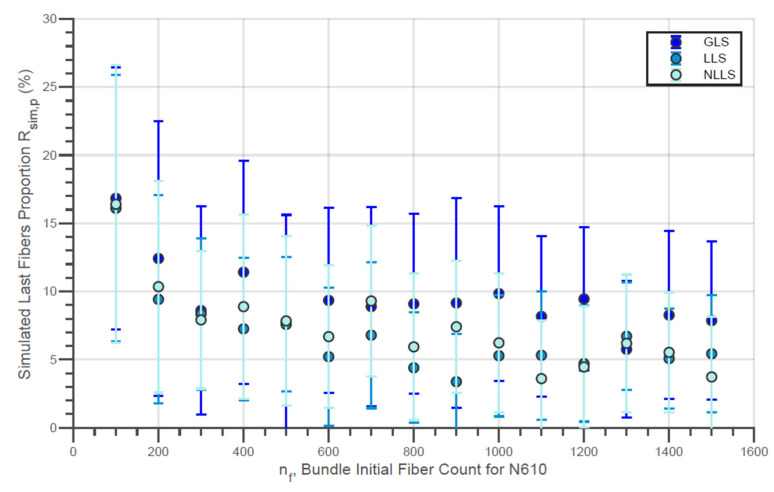
Simulated Last Fiber Proportion for Different Fiber Amounts for Virtual N610 Fiber Bundles.

**Figure 17:**
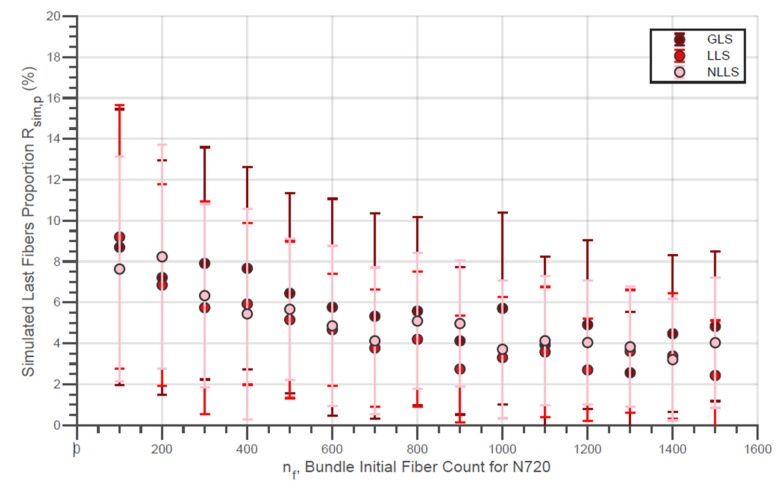
Simulated Last Fiber Proportion for Different Fiber Amounts for Virtual N720 Fiber Bundles.

In addition, the increase in the value of $\sigma_{sim,LF}$ verified in Figure 12 and Figure 15 with the initial amount of fibers is also due to the negative variation in the proportion of the last fibers within the simulated bundle $R_{sim,p}$. This tendency can be seen for the virtual fibers N610 and N720, for all the different models of load sharing, in Figure 16 and Figure 17.

In addition, the ΔL load increment also plays a very important role in the simulation. As mentioned above, this parameter was experimentally determined by load diagrams for both N610 and N720 single fibers and applied to the simulations. In experimental terms, the ΔL increment represents the rate at which the load is applied to the bundle. Now, let $\delta L_{break}$ be the average difference of individual breaking loads of the fibers within the virtual bundle. If $\left(\Delta L\times n_{f}^{-1}\right)\approx \delta L_{break}$, with $\Delta L\times n_{f}^{-1}$ being the average individual applied load, a large amount of fibers breaks within a load increment. With this, lower external loads L are needed for the total rupture, and lower bundle strength and strength of fibers within the bundle are registered.

On the other hand, smaller load increments ΔL result in a slower and more detailed simulation with lower fiber breakage per simulation step. With this, a smaller number of fibers will be present in the final breaking event even if the breaking load is not altered. In this way, both the bundle strength and the individual fiber strength will be higher. This fact suggests that the load rate plays a key role in the bundle true strength. However, further work is still needed to generate experimental data to confirm this hypothesis.

### 3.4. Discussion

The true strength of the N610 and N720 fiber bundles was obtained through experiments and simulations. Although the results were obtained through tests oriented in different ways, it is still possible to observe some similarity between the results.

Initially, the bundle true strength $\sigma_{b,true}$ determined through the method of counting rupture signals in loading ranges (Figure 1) increase with $n_{f}$ the same way as the simulation for the different load-sharing models employed (Figure 10 and Figure 13). As mentioned, it is possible that calculated $\sigma_{b,true}$ parameters are overestimated due to the limitation of the present method of considering equal amounts of broken fibers for each ΔF<0. Likewise, it is natural that results presented by the simulations are higher compared to the experiments since external factors (e.g., not loading all fibers at the beginning of the test, lack of alignment, slipping, etc.) were not considered. However, for a better conclusion, the method of counting signals needs to be improved, and the simulations must be performed with variable ΔL according to the amount of fibers present in the bundle. Still, it is also possible that the simulations allow us to better define the variable width of the loading ranges in improving the process of counting rupture signals.

In addition, the ΔL used for the simulations was determined through experiments performed with the filaments as mentioned in the materials and methods section. The ΔL used for the N610 was higher than that for the N720, which gives to the N720 simulations a higher resolution compared to the N610. As mentioned in the last paragraph, it is also expected that fewer fibers break with each load increase for simulations where ΔL is smaller. Thus, it is understandable that the results presented in Figure 16 and Figure 17 are different and that, even the $R_{sim,p}$ for N720 bundles is lower than that for N610 bundles. Based on this fact, for a more accurate comparison between $R_{p}$ and $R_{sim,p}$, simulations with different ΔL would be necessary for the different $n_{f}$ values considering that the loading rate is higher for more numerous beams in a strain-piloted test. Since the simulation represented a force-piloted test, the experimental and simulation results for $R_{p}$ and $R_{sim,p}$ are, in general, not comparable.

Finally, it was also verified that, although the filament presents greater strength in comparison to bundles, the Weibull modulus of bundles is considerably higher than that of the filaments. In general, this denotes that the bundle has a less wide rupture strength distribution than filaments. On the other hand, such a parameter is approximately constant within $n_{f}$ and not so different (sometimes even smaller) than that of simple fibers (Figure 8 and Figure 9). According to the results presented in the simulations (Figure 11 and Figure 14), this parameter is not really affected by $n_{f}$, but, like the Weibull modulus of engineering strength, it should be higher compared to the filaments. For this difference, two explanations can be associated: the calculation of the true strength by means of the signal break count does not take into account the possibility of a greater number of fibers broken in higher loading ranges (which, in addition to overestimating the parameter, also generates a wider distribution (lower Weibull modulus) and/or the simulation overestimates this value by not considering adverse factors, resulting in true strengths not so widely distributed. In any case, it is expected that a high Weibull filament modulus will result in bundles with strengths more concentrated around their characteristic value.

## 4. Conclusions and Further Comments

In this work, experimental tests were performed with fiber bundles of different size/fiber counts. The results obtained through the presented procedure confirm that the true strength of bundles cannot be estimated just by adding the strength of individual fibers, e.g., $\sigma_{bundle}\neq n_{f}\cdot \sigma_{fiber}$. In addition, the experiments together with the employed method demonstrate that the initial amount of fibers in the bundle influences the true strength of bundles of both N610 and N720 fibers. However, due to the limitation presented by the procedure, it is possible that it overestimates the true strength of the fiber bundle due to the underestimation of the amount of fibers surviving at the time of the final rupture. On the other hand, an alternative to solve this problem would be to consider progressively larger loading ranges for counting rupture signals. This approach is currently under development as a result of this project. Still, by other calculation methods, it was found that the true strength of bundles is constant although there is no consensus on which of the models employed is the most appropriate. Regardless, the values of the fraction of fibers present at the moment of rupture were also shown to be different among the approaches used. In any case, through strict experiments it was found that such a fraction is less than that predicted by the theoretical values. This fact reinforces the assumption that stronger fibers may break previously when reaching maximum load and that the bundle true strength may be higher than the strength of the filaments, although the bundle engineering strength is lower.

In addition, a numerical procedure was developed and executed to evaluate the true strength of fiber bundles based on force-piloted simulation. For this, load-sharing models of fibers in a bundle using global and local approaches as well as hybrid versions of these, e.g., nonlocal load sharing, were implemented. Unfortunately, due to the statistical values of the input data, e.g., the strength distribution of individual fibers, the different load-sharing models resulted in overlapping distribution functions, preventing a clear assignment. Regardless, the simulations, as well as the experiments with the rupture signal processing, show that the true strength of the bundles is higher for samples with larger initial amounts of fibers. The simulations also help confirm that, under a non-global loading distribution, some of the strongest fibers in the bundle will break before maximum loading. In addition, the simulations suggest that the range of instants in which the ruptures tend to occur is also influenced by the initial amount of fibers in the bundle. This fact is possibly associated with the variation of the loading rate in the remaining fibers of the bundle during the tests. Based on this, new tests under different initial loading rates can be done to verify this hypothesis and also to point out which load-sharing model is the most suitable.

Finally, it is noted through the method used in the experiments and through the simulations that both the true strength of bundles and the last fiber proportion may be influenced in some way by $n_{f}$. However, further refinement is needed in the method of determining the true strength of the bundles presented in this work. For example, simulations with different ΔL for different $n_{f}$ can be performed to better approximate the results of the experiments and simulations.

## Figures and Tables

**Table 1:** Typical properties of N610 and N720 single fibers [17].

Property	Units	N610	N720
Chemical composition	wt.%	>99 Al_2_O_3_	85 Al_2_O_3_ 15 SiO_2_
Filament diameter	μm	10–12	10–12
Denier/Nominal filament count	g·9000 m^−1^	1500/400 3000/750 10,000/2550	1500/400 3000/750
Crystal size	nm	<500	<500
Crystalline phase		α-Al_2_O_3_	α-Al_2_O_3_ + Mullite
Density	g·cm^−3^	3.90	3.40
Filament tensile strength (25.4 mm gauge)	MPa	3100	2100
Filament tensile modulus	GPa	380	260

**Table 2:** Number of replications for the fiber bundle tests.

**N610**						
**Initial Amount of Fibers within the Bundle**	**100**	**200**	**300**	**400**	**750**	**1500**
Test replications	42	42	44	35	15	21
**N720**						
**Initial Amount of Fibers within the Bundle**	**100**	**200**	**300**	**400**	**800**	
Test replications	42	40	40	49	15	

**Table 3:** Engineering strength experimental results for the fiber bundles.

**N610**						
**Initial Amount of Fibers within the Bundle**	**100**	**200**	**300**	**400**	**750**	**1500**
Characteristic Maximal Force $F_{max}$ (N)	11.02	20.88	34.32	43.47	91.89	145.49
Characteristic Engineering Strength $\sigma_{eng}$ (MPa)	1160.2	1098.8	1203.9	1143.6	1289.5	1020.7
Weibull Modulus m	1.84	3.67	4.15	5.13	5.35	3.74
**N720**						
**Initial Amount of Fibers within the Bundle**	**100**	**200**	**300**	**400**	**800**	
Characteristic Maximal Force $F_{max}$ (N)	4.58	14.80	16.55	25.50	49.56	
Characteristic Engineering Strength $\sigma_{eng}$ (MPa)	481.5	778.5	580.5	670.8	651.9	
Weibull Modulus m	2.28	4.85	3.38	2.88	6.26	

**Table 4:** Average rupture signal amounts and standard deviation for the different fiber types and initial amount of fibers.

**N610**						
**Initial Amount of Fibers within the Bundle**	**100**	**200**	**300**	**400**	**750**	**1500**
Average amount of rupture signals	24 ± 13	36 ± 14	36 ± 13	75 ± 25	51 ± 36	88 ± 43
**N720**						
**Initial Amount of Fibers within the Bundle**	**100**	**200**	**300**	**400**	**800**	
Average amount of rupture signals	25 ± 9	38 ± 13	38 ± 10	42 ± 17	106 ± 42	

**Table 5:** Characteristic engineering strength and Weibull moduli for the N610 and N720 samples considering different conditions.

**N610**						
**Initial Amount of Fibers within the Bundle (** $n_{f}$ **)**	**100**	**200**	**300**	**400**	**750**	**1500**
Lamon’s et al. Characteristic Tow Strength (MPa) ($\sigma_{F,610}$) $\alpha_{c,610}=38.17\%$	3107.6	2721.4	2709.0	2727.7	3632.5	1841.8
Lamon’s et al. Characteristic Tow Strength (MPa) ($\sigma_{F,610}$) $\alpha_{c,610}=8.40\%$ (with Wilson’s $m_{s,610}$)	2127.7	1765.5	1914.8	1870.0	2081.0	1252.5
Characteristic True Strength (MPa) $\sigma_{true,610}^{*}$ Considering γ	4801.1	3884.8	3325.0	5425.9	4376.1	5398.7
	$\gamma_{avg}$ 42.27%	$\gamma_{avg}$ 33.16%	$\gamma_{avg}$ 30.34%	$\gamma_{avg}$ 31.77%	$\gamma_{avg}$ 31.12%	$\gamma_{avg}$ 12.61%
**N720**						
**Initial Amount of Fibers within the Bundle (** $n_{f}$ **)**	**100**	**200**	**300**	**400**	**800**	
Lamon’s et al. Characteristic Tow Strength (MPa) ($\sigma_{F,720}$) $\alpha_{c,720}=49.12\%$	1532.8	2076.0	1619.0	1511.0	1536.5	
Lamon’s et al. Characteristic Tow Strength (MPa) ($\sigma_{F,720}$) $\alpha_{c,720}=12.33\%$ (with Wilson’s $m_{s,720}$)	993.3	1150.6	873.3	1033.6	813.9	
Characteristic True Strength (MPa) $\sigma_{true,720}^{*}$ Considering γ	1887.3	2494.9	1768.1	2507.8	1294.3	
	$\gamma_{avg}$ 42.81%	$\gamma_{avg}$ 22.40%	$\gamma_{avg}$ 26.54%	$\gamma_{avg}$ 24.65%	$\gamma_{avg}$ 8.70%	

**Table 6:** Final fraction of fibers obtained through different methods.

**N610**						
**Initial Amount of Fibers within the Bundle (** $n_{f}$ **)**	**100**	**200**	**300**	**400**	**750**	**1500**
$R_{p,610}$ (%)	33.10	34.17	38.69	28.80	34.61	17.21
$R_{p,610}^{*}$ (%) Considering γ	18.67	23.25	29.99	18.34	26.11	18.98
$\left(1-\alpha_{c}\right)\left(1-\gamma_{avg}\right)$ $\alpha_{c,610}=38.17\%$ (%)	35.69	41.33	43.07	42.19	42.59	54.03
$\left(1-\alpha_{c}\right)\left(1-\gamma_{avg}\right)$ $\alpha_{c,610}=8.40\%$ (%) (with Wilson’s $m_{s,610}$)	52.88	61.23	63.81	62.50	63.09	80.04
$\left(1-\alpha_{alt}\right)$ (%)	22.60	22.19	24.03	23.25	25.96	20.28
	$\gamma_{avg}$ 42.27%	$\gamma_{avg}$ 33.16%	$\gamma_{avg}$ 33.34%	$\gamma_{avg}$ 31.77%	$\gamma_{avg}$ 31.12%	$\gamma_{avg}$ 12.61%
**N720**						
**Initial Amount of Fibers within the Bundle (** $n_{f}$ **)**	**100**	**200**	**300**	**400**	**800**	
$R_{p,720}$ (%)	33.43	32.07	32.81	33.05	17.61	
$R_{p,720}^{*}$ (%) Considering γ	19.02	24.84	24.02	24.79	16.04	
$\left(1-\alpha_{c}\right)\left(1-\gamma \right)$ $\alpha_{c,720}=49.12\%$	29.09	39.48	37.37	38.33	46.45	
$\left(1-\alpha_{c}\right)\left(1-\gamma \right)$ $\alpha_{c,720}=12.33\%$ (with Wilson’s $m_{s,720}$)	50.14	68.03	64.40	66.06	80.04	
$\left(1-\alpha_{alt}\right)$ (%)	20.48	34.32	25.04	28.94	29.17	
	$\gamma_{avg}$ 42.81%	$\gamma_{avg}$ 22.40%	$\gamma_{avg}$ 26.54%	$\gamma_{avg}$ 24.65%	$\gamma_{avg}$ 8.70%	

**Table 7:** Average Weibull modulus for the True Strength of N610 and N720 virtual bundles under different load-sharing models.

	N610 Weibull Modulus for True Strength ($n_{f}$ from 100 to 1500), Average	N720 Weibull Modulus for True Strength ($n_{f}$ from 100 to 1500), Average
GLS model	4.91 ± 0.98	3.96 ± 0.45
LLS model	3.89 ± 0.58	3.57 ± 0.50
NLLS model	4.10 ± 0.57	3.58 ± 0.39

## Data Availability

The raw/processed data required to reproduce these findings cannot be shared at this time as the data also form part of an ongoing study.
